# Irisin Controls Growth, Intracellular Ca^2+^ Signals, and Mitochondrial Thermogenesis in Cardiomyoblasts

**DOI:** 10.1371/journal.pone.0136816

**Published:** 2015-08-25

**Authors:** Chao Xie, Yuan Zhang, Tran D. N. Tran, Hai Wang, Shiwu Li, Eva Vertes George, Haoyang Zhuang, Peilan Zhang, Avi Kandel, Yimu Lai, Dongqi Tang, Westley H. Reeves, Henrique Cheng, Yousong Ding, Li-Jun Yang

**Affiliations:** 1 Department of Pathology, Immunology, and Laboratory Medicine, University of Florida College of Medicine, Gainesville, FL, 32610, United States of America; 2 Center for Stem Cell & Regenerative Medicine, The Second Hospital of Shandong University, Jinan, 250012, P. R. China; 3 Department of Comparative Biomedical Sciences, School of Veterinary Medicine, Louisiana State University, Baton Rouge, LA, 70803, United States of America; 4 Department of Cell Biology and Anatomy, University of South Carolina of Medicine, Columbia, SC, 29209, United States of America; 5 Department of Medicine, University of Florida College of Medicine, Gainesville, FL, 32610, United States of America; 6 Department of Medicinal Chemistry, Center for Natural Products, Drug Discovery and Development, College of Pharmacy, University of Florida, Gainesville, FL, 32610, United States of America; University of Cincinnati, College of Medicine, UNITED STATES

## Abstract

Exercise offers short-term and long-term health benefits, including an increased metabolic rate and energy expenditure in myocardium. The newly-discovered exercise-induced myokine, irisin, stimulates conversion of white into brown adipocytes as well as increased mitochondrial biogenesis and energy expenditure. Remarkably, irisin is highly expressed in myocardium, but its physiological effects in the heart are unknown. The objective of this work is to investigate irisin’s potential multifaceted effects on cardiomyoblasts and myocardium. For this purpose, H9C2 cells were treated with recombinant irisin produced in yeast cells (r-irisin) and in HEK293 cells (hr-irisin) for examining its effects on cell proliferation by MTT [3-(4, 5-dimethylthiazol-2-yl)-2,5-diphenyltetrazolium bromide] assay and on gene transcription profiles by qRT-PCR. R-irisin and hr-irisin both inhibited cell proliferation and activated genes related to cardiomyocyte metabolic function and differentiation, including myocardin, follistatin, smooth muscle actin, and nuclear respiratory factor-1. Signal transduction pathways affected by r-irisin in H9C2 cells and C57BL/6 mice were examined by detecting phosphorylation of PI3K-AKT, p38, ERK or STAT3. We also measured intracellular Ca^2+^ signaling and mitochondrial thermogenesis and energy expenditure in r-irisin-treated H9C2 cells. The results showed that r-irisin, in a certain concentration rage, could activate PI3K-AKT and intracellular Ca^2+^ signaling and increase cellular oxygen consumption in H9C2 cells. Our study also suggests the existence of irisin-specific receptor on the membrane of H9C2 cells. In conclusion, irisin in a certain concentration rage increased myocardial cell metabolism, inhibited cell proliferation and promoted cell differentiation. These effects might be mediated through PI3K-AKT and Ca^2+^ signaling, which are known to activate expression of exercise-related genes such as follistatin and myocardin. This work supports the value of exercise, which promotes irisin release.

## Introduction

Regular exercise is a cornerstone in the prevention and treatment of chronic metabolic diseases, cardiovascular disease, and aging-related muscle wasting (sarcopenia) [1, 2]. Both aerobic (endurance) and resistance (strength) exercise reduce cardiovascular risk profile and increase basal metabolic rate. Myokines released from muscle during exercise mediate exercise associated benefits [3] by communicating with other tissue/organs and exerting metabolic effects in an autocrine, paracrine, and/or endocrine manner [4].

Irisin is a recently discovered exercise-induced myokine that has received considerable attention due to its promising effects in mediating health-related benefits of physical activity [5]. Irisin is a proteolytic product of fibronectin type III domain containing 5 (FNDC5) transmembrane protein whose expression is induced by exercise training via up-regulation of peroxisome proliferator-activated receptor (PPAR)-γ co-activator 1α (PGC-1α) [5]. PGC-1α interacts with a broad range of transcription factors to modulate various biological responses such as glucose/fatty acid metabolism and heart development [6, 7]. After exercise, overexpressed PGC-1α drives the expression of uncoupling protein 1 (UCP1), nuclear respiratory factor (NRF) and its downstream target mitochondrial transcription factor A (TFAM), which controls the process of mitochondrial biogenesis [6]. We and others have shown that recombinant irisin (r-irisin) causes browning of white adipose cells and reduces the body weight of obese mice via extracellular signal—related kinase (ERK) and p38 protein kinase (MAPK) signaling [5, 8]. The effects of irisin *in vitro* and *in vivo* suggest that this molecule may be useful for preventing and treating obesity.

Although the physiological role of irisin in humans and other species is largely unknown and controversial [9], FNDC5 has been detected in many tissues in addition to skeletal muscle such as myocardial and smooth muscles, endothelium, brain, adipose tissue, liver, kidney and pancreas [10, 11]. This fact may suggest multiple functions of irisin. Strikingly, cardiac muscle expresses a high level of FNDC5 and after exercise produces more irisin than skeletal muscle [10]. The high level of irisin in cardiac muscle suggests its potential but poorly-explored roles in cardiac function and performance [12–14]. In addition, human studies have indicated a tight association of irisin with heart health [15, 16]. However, the molecular mechanism by which irisin signals remains unknown.

Here, we sought to characterize irisin’s effects and to elucidate its mechanism of action at the cellular and whole animal levels. H9C2 cells are known to have electrophysiological and biochemical properties of cardiac tissues [17], and were chosen as the cellular model for our studies. We found that r-irisin inhibited cardiomyoblast (H9C2) cell proliferation and activated genes related to metabolic function/differentiation. R-irisin treatment of H9C2 cells also activated the PI3K-AKT and Ca^2+^ signaling pathways and enhanced mitochondria thermogenesis. Furthermore, we detected the activation of multiple signaling pathways in myocardium after injecting r-irisin into mice. Finally, our results suggested the existence of an irisin-specific receptor on the membrane of H9C2 cells. These findings established irisin as a critical modulator of cardiomyoblast performance and provided novel insights into the understanding of irisin’s relationship with heart health and cardiovascular disease (CVD).

## Materials and Methods

### Cell Culture

The embryonic rat cardiomyoblast cell line H9C2 and human embryonic kidney cell line 293 (HEK 293 cells) were purchased from American Type Culture Collection (Manassas, VA) and cultured in Dulbecco’s Modified Eagle’s Medium (DMEM) (Coning cellgro, 10-013-CV) containing 10% fetal bovine serum (FBS) (Atlanta Biologicals) and 100 U/mL penicillin/streptomycin at 37°C in a 5% CO_2_ incubator. H9C2 cells were passaged every 4 days at a ratio of 1:3 and the subcultivation ratio of HEK 293 cells was 1:6 weekly.

### Production of Recombinant Irisin in Yeast and Mammalian Cells

R-irisin was expressed and purified from *Pichia pastoris* cultures as previously described [8]. *P*. *pastoris* was also transformed with an empty expression vector and the supernatant of transformed cells was prepared as a negative control [8]. His-tagged r-irisin (r-irisin-his) plasmid was constructed by adding six His codons to the 3’-end of irisin. The construct was expressed in *P*. *pastoris* cultures for protein purification with affinity column as previously described [18, 19]. To produce r-irisin in human HEK 293 cells (named hr-irisin), its cDNA was first amplified with the primers 5’-GAATTC CTCAACCATGGAATTCTCTCCATCCGCTCC-3’ and 5’-CTCGAGCATGGTTGAGCTCGAGATCTCAGTCCGG-3’ in PCR reactions. The purified PCR product was cloned into the pSectTag2/HygroA vector under the control of IgK signal peptide. As a negative control, the green fluorescent protein (GFP) gene was cloned into the same site of the above vector. The resulting construct was transfected into HEK 293 cells using Lipofectamine 2000 (Invitrogen, 11668019) following the manufacturer’s protocols. Transfected HEK 293 cells were cultured for 4 days, and hr-irisin was secreted into the culture medium. Harvested supernatant was dialyzed and concentrated to obtain hr-irisin. Recombinant irisin from both sources was assayed by western blotting with anti-irisin antibody (see below for details), and stored at -80°C without further purification. Culture medium of HEK 293 cells transfected with GFP expression plasmid was processed as a negative control.

### Preparation of Anti-Irisin Rabbit Polyclonal Antibody

The purified r-irisin was used as the antigen to raise a polyclonal antibody in New Zealand White rabbits according to a previous strategy [20]. In brief, rabbits were subcutaneously immunized in multiple subcutaneous locations with 20μg/mL of purified r-irisin emulsified in Complete Freund’s Adjuvant. Two subsequent booster immunizations 21-days apart were given subcutaneously in two locations using 10 μg/mL of r-irisin emulsified in Incomplete Freund’s Adjuvant. Prior to the first immunization, pre-immune serum samples were obtained from the central auricular artery of rabbits as negative control. Post-immunization serum samples (test bleed) were obtained 7 days after the 3rd immunization. The specificity and working dilution of the irisin antibody were determined by western blotting. A final blood sample was collected under anesthesia, and serum was aliquoted and stored at -80°C.

### Determination of H9C2 Proliferation

To determine the time course of irisin action, H9C2 cells (1000 cells/well) seeded in 96-well plates (Costar, 3595) were treated with hr-irisin, 50 nM of r-irisin, or control reagent for 0–4 days. In dose-response studies, the cells were cultured in the presence of 6 to 100 nM of r-irisin or control for 4 days. Cell proliferation at each time point and under different concentrations was then quantitated by 3-(4, 5-dimethylthiazol-2-yl)-2, 5-diphenyltetrazolium bromide (MTT) colorimetric assay following the manufacturer’s protocol. Briefly, 10 μl of 5 mg/ml MTT solution (Sigma-Aldrich) was added to each well, and samples were incubated at 37°C. After 4 hours, the samples were centrifuged, and the absorbance of supernatants was measured at 570 nm using the UV/vis microplate spectrophotometer (BioTeK). The MTT assay was performed with 16 replicates in three independent experiments.

### Quantitative Real Time Polymerase Chain Reaction (qRT-PCR)

H9C2 cells were seeded in 6 wells plates (Costar, 3516) and treated with r-irisin (50 nM) or hr-irisin for 6 h and 24 h. The total RNA was then extracted from H9C2 cells using the TRIzol kit (Invitrogen, 15596–026). Isolated total RNA (2 μg) was reverse-transcribed and amplified with the TaqMan kit (Applied Biosystems) according to manufacturer’s protocols. The real time-qPCR reactions were performed on an RT-PCR machine with SsoFast EvaGreen Supermix (Bio-Rad). PCR primers were designed and synthesized by Integrated DNA Technologies (Coralville, IA). Primer sequences were listed in S1 Table.

### Western Blotting and Quantitation

H9C2 cells in 6-well plates were switched to serum free DMEM for 2 h and then treated with r-irisin (50 nM) or negative control for 0, 5, 20 and 30 minutes. At each time point, cells were collected and washed twice with PBS. Cell lysates were prepared by incubating cells with RIPA cell lysis buffer on ice. Cellular protein in all samples was measured using DC Protein Assay (Bio-Rad, 500–0112). Equal amounts of protein from all samples were loaded into each well of a 12% SDS-polyacrylamide gel. After electrophoresis, proteins were transferred to PVDF membranes and blotted with the indicated primary antibodies (rabbit anti-irisin, 1:1000; anti-ERK1/2, anti-phospho-ERK1/2, anti-p38, anti-phospho-p38, anti-AKT, anti-phospho-AKT, anti-STAT3, anti-phospho-STAT3, 1:1000, Cell Signaling; rabbit anti-β-actin antibody, 1:10000, Sigma—Aldrich) with gentle agitation at 4°C overnight. After being washed with TBST, the membranes were then incubated with HRP-conjugated secondary antibodies at room temperature for 1 h. Immune complexes were visualized using the ECL method and immune-reactive bands quantitated by densitometry using an Alpha Imager 2200.

### Calcium Imaging Analysis

H9C2 cells grown on round glass coverslips were loaded with 5 μM Fura-2 AM at 37°C for 30 min and mounted inside a closed system perfusion chamber. Ca^2+^ imaging buffer containing 10 mM HEPES (pH 7.3), 136 mM NaCl, 4.8 mM KCl, 1.2 mM CaCl_2_, 1.2 mM MgSO_4_, 4 mM glucose, and 0.1% BSA was used for Fura-2 AM loading and perfusion. Calcium measurements were obtained with a dual excitation fluorometric imaging system (TILL-Photonics, Gräfelfingen, Germany) controlled by TILLvisION software. Fura-2 AM loaded cells were excited at wavelengths of 340 nm and 380 nm. Fluorescence emissions were sampled at a frequency of 1 Hz and expressed as a relative ratio of the fluorescence intensity at different wavelengths (F340/F380). The single cell traces with the averages for all cells were plotted for each r-irisin concentration along with the peak Ca^2+^ increases.

### Mitochondrial Flux Analyses

The XF96 Extracellular Flux Analyzer (Seahorse Bioscience, Billerica, MA) was used to measure the extracellular flux changes of oxygen in H9C2 cell culture supernatants. The cells were plated at 15,000 cells per well into XF96 microplates and treated with 25 to 400 nM of r-irisin or control for 72 h. Prior to the assay, cells were switched to unbuffered DMEM supplemented with 1 mM pyruvate and cultured 1 h at 37°C in a non-CO_2_ incubator. The cartridge in the XF96 Analyzer was calibrated followed by measurement of OCR (oxygen consumption rate) from the cell plate. Three initial measurements of OCR were made using cycles comprised of 2-min mixing and 3-min measurement. Injection of oligomycin (1 μM) was used to measure the ATP linked OCR. Oligomycin A is an inhibitor of ATP synthase. The inhibition of ATP synthesis by oligomycin A is expected to significantly reduce electron flow through the electron transport chain. OCRs were further measured by sequentially adding the mitochondrial uncoupler FCCP (Carbonyl cyanide-4-(trifluoromethoxy)phenylhydrazone) (0.75 μM) to determine maximal respiration and rotenone (1 μM) to determine the non-mitochondrial respiration. Experimental treatments were performed on 8 wells of each plate as technical replicates and each experiment had 3 biological replicates. Values were normalized to the total number of cells/well.

### Activation of Signaling Pathways by R-Irisin in Mouse Hearts

Male C57BL/6 mice of 8-week-old were purchased from Shanghai Slac Laboratory Animal Co., Ltd and housed in the animal care facility in 12-h light, 12-h dark cycles and fed ad libitum with normal chow. Mice were intraperitoneal injected with purified r-irisin at a dose of 10 μg/g of body weight. Control mice were received the supernatant of yeast transformed with an empty expression vector at the same volume. Then mice were anesthetized by intraperitoneal injection of ketamine and xylazine and the hearts were perfused with 20 mL saline via the inferior vena cava before being removed from body. All of the animal protocols were conducted in accordance with the NIH Guide for Care and Use of Laboratory Animals and were approved by the Institutional Animal Care and Use Committee at Shandong University, China(Permit Number: ECAESDUSM2012093). The left ventricle was collected at different time points (0, 30, 60, 90, 120 minutes) following r-irisin injection (n = 3/group) and proteins in heart tissues were harvested for western blotting analysis.

### Flow Cytometry

H9C2 cells were released by trypsin and then incubated with different concentrations of r-irisin-his or r-irisin for 60 min at room temperature. The cells were then incubated with anti-his-phycoerythrin (PE) (Miltenyi Biotec) or isotype controls (30 min 22°C), washed, and resuspended in PBS/0.5% BSA. Fifty thousand events per sample were acquired using a BD LSRII flow cytometer (BD Bioscience, San Jose, CA) and analyzed with FCS Express 3 (De Novo Software, Ontario, Canada).

### Statistical Analyses

Results were presented as mean ± SE of at least three independent experiments. Each experiment was conducted in triplicate. Statistical significance among multiple groups was analyzed by one-way ANOVA followed by Student’s t-test for comparison of the results between two groups using Prism 5 (Graphpad Software, Inc). *, **, and *** indicates p < 0.05, p <0.01, and p < 0.001 statistical differences compared to control, respectively.

## Results

### R-Irisin Regulated Growth in Rat Cardiomyoblasts

**Fig 1:**
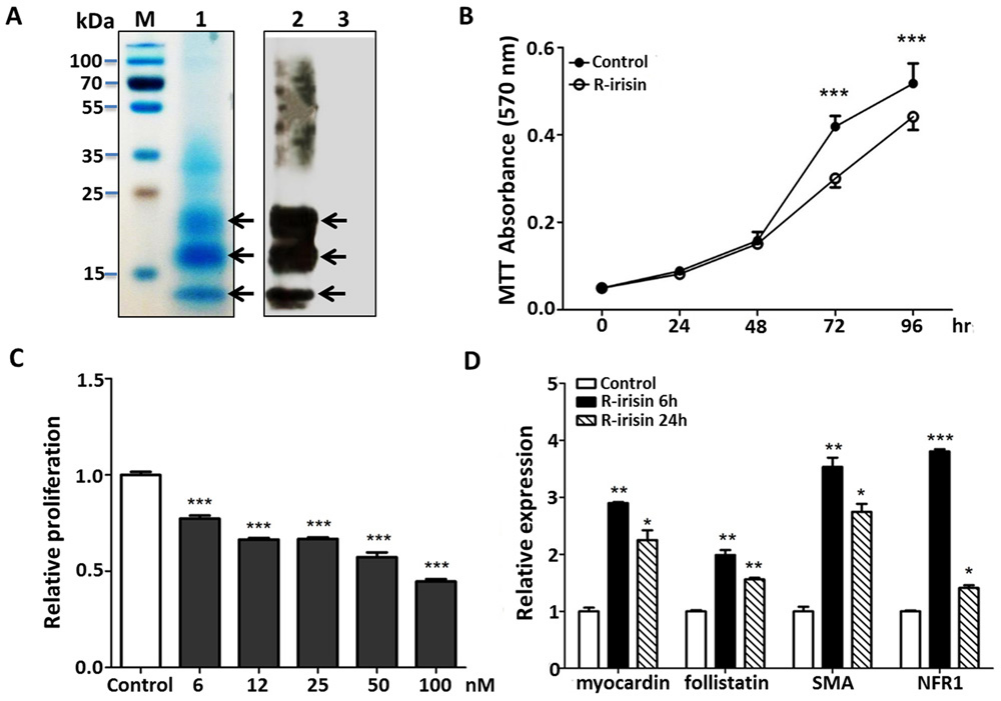
H9C2 growth responses to r-irisin treatment. A: SDS-PAGE and western blotting analysis of human irisin expressed in *Pichia pastoris*. *P*. *pastoris* culture supernatant was harvested and analyzed by Coomassie blue stained SDS-PAGE (Lane1) and western blotting (Lane 2). The negative control showed no irisin in western blotting analysis (Lane 3). B: The time response of H9C2 cells to r-irisin (50 nM) treatment. Cell proliferation was measured by MTT assay. C: The dose response of H9C2 cells to r-irisin 3 days treatment. D: Expression profile of selected genes in H9C2 cells treated with r-irisin (50 nM) at different time points. The gene transcription was measured by quantitative RT-PCR, and compared to the expression level of β-actin. The data was shown as mean ±SD of three independent experiments. (*, **, and *** indicating *p* < 0.05, *p* <0.01, and *p* < 0.001 statistical differences compared to control, respectively.)

As previously described, we expressed the r-irisin protein in *P*. *pastoris* [8]. SDS-PAGE analysis of culture supernatant of transformed yeast cells (Fig 1A, lane 1) revealed three major bands with molecular weights of ~12, 17 and 22 kDa. The identities of these bands were further characterized by western blotting with anti-irisin rabbit polyclonal antibody (Fig 1A, lane 2). The negative control (supernatant from yeast transformed with empty vector) showed no r-irisin products in western blotting (Fig 1A, lane 3). Human irisin can be post-translationally modified with sugar moieties at two distinct locations (Asn7 and Asn52) [8] and these bands presumably represent differential glycosylation of r-irisin. The bioactive r-irisin expressed in yeast for these studies was about 95% pure based on density analysis.

Irisin was detected in cardiac muscle of rat and its correlation with human CVD has been reported [12–15]. However, its physiological role in cardiac function remains unexplored. We first examined the effect of r-irisin on growth of rat cardiomyoblasts. H9C2 cells were treated with r-irisin (50 nM) or negative control, and cell proliferation after 0, 24, 48, 72, and 96 h was determined by MTT assay (Fig 1B). Compared with the negative control, r-irisin (50 nM) significantly inhibited H9C2 cell proliferation after 72-h treatment. The inhibitory effect was dose-dependent and significant even at low concentrations (6–12 nM) (Fig 1C). To examine irisin-mediated changes in gene transcription, we treated H9C2 cells with r-irisin (50 nM) or negative control, and then determined the expression of myocardium-related genes using qRT-PCR after 6 h and 24 h. As shown in Fig 1D, r-irisin upregulated the expression of the following genes at 6 h: myocardin, follistatin, SMA, and NRF1. In contrast, up-regulation was much lower at 24 h. Myocardin [21, 22], Follistatin [23], and SMA [24] are involved in cardiomyocyte growth and differentiation, and their overexpression suggests a role of irisin in cardiomyoblast growth. In addition, irisin may enhance mitochondrial biogenesis via NRF1 [25].

### R-Irisin Activated PI3K-AKT Signaling Pathway

**Fig 2:**
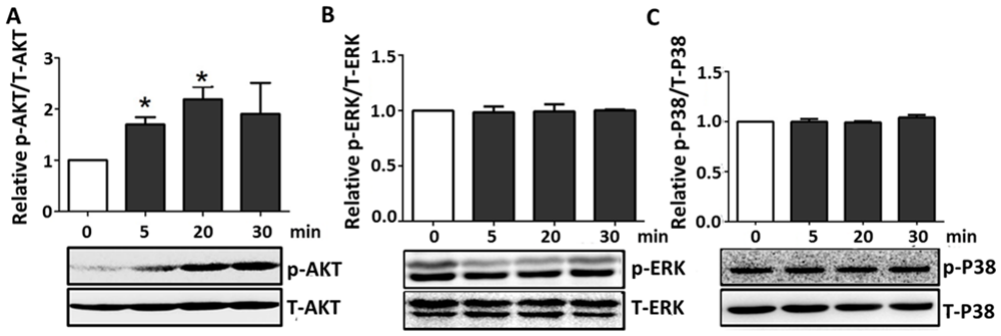
Activation of different signaling pathways in H9C2 cells after r-irisin treatment. H9C2 cells were treated with r-irisin (50 nM). After 0, 5, 20, and 30 min, protein was collected for western blotting. Total β-actin or AKT served as protein-loading control. Bottom is typical blots; the top bar graphs represent densitometry analyses for p-AKT/AKT (A), p-ERK/β-actin (B) and p-p38/β-actin (C). All data were normalized against the control group. Results represent the means from three independent experiments. (* indicating *p* < 0.05 statistical differences compared to control, respectively.)

Heart development, growth, and health are regulated by multiple signaling pathways. AKT is a serine/threonine protein kinase that regulates a variety of cellular functions in different tissues, and is essential during postnatal cardiac development. Mitogen-activated protein (MAP) kinases including ERK1/2 and p38 also have been implicated in different aspects of cardiac regulation, from development to pathological remodeling. Given their importance for cardiac development, growth, and physiology [26–28], we evaluated the extent to which irisin regulates the MAPK/ERK, p38-MAPK, and PI3K-AKT signaling pathways. After the treatment of r-irisin (50 nM) for 5, 20, or 30 min, H9C2 cells were lysed for western blotting analysis using antibodies specific to phosphorylated AKT, ERK and p38. Phosphorylation of AKT (on threonine 308) was observed after 5 min r-irisin treatment and peaked at 20 min (Fig 2A). In contrast, over the entire r-irisin treatment period, no significant activation of the ERK- and p38-MAPK signaling pathways was detected (Fig 2B and 2C). These data demonstrated that the PI3K-AKT pathway was involved in the early response to irisin signaling.

### R-Irisin Increased Intracellular Ca^2+^ Concentration

**Fig 3:**
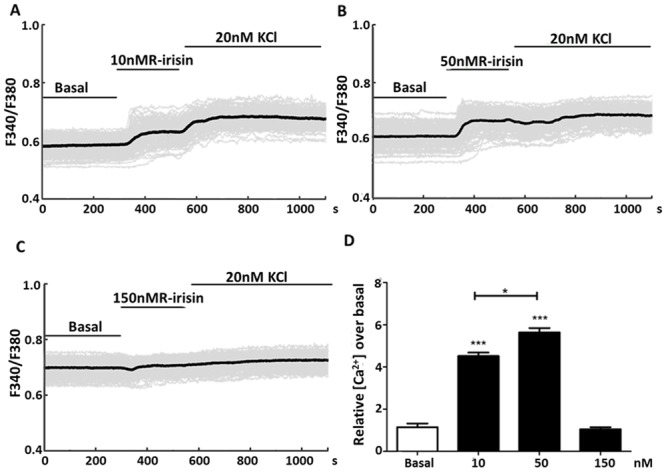
Stimulation of H9C2 cells with r-irisin increased intracellular Ca^2+^ concentration. Real-time Ca^2+^ imaging analysis was used to measure intracellular Ca^2+^ levels in H9C2 cells after stimulation with low (10 nM, A), intermediate (50 nM, B), and high (150 nM, C) r-irisin concentrations, followed by 20 mM KCl treatment. Gray lines = single cell traces; Black line = average of all cells. D: The average Ca^2+^ peak by each r-irisin concentration compared to basal levels prior to stimulation. Values represent the mean ± SD from 95–113 cells per concentration. (*, **and *** indicating *p*<0.05, *p*<0.01, and *p*<0.001 statistical differences compared to control, respectively.)

Elevated intracellular Ca^2+^ concentrations ([Ca^2+^]_i_) are critical for myocardial function and maintenance of excitation—contraction coupling, and exercise is known to stimulate Ca^2+^ in cardiomyocytes [29]. We hypothesized that irisin’s effects on H9C2 is exerted by increasing [Ca^2+^]_i_. To test this hypothesis, we performed real-time Ca^2+^ imaging analysis in H9C2 cells during stimulations with low (10 nM), intermediate (50 nM), and high (150 nM) concentrations of r-irisin. Compared with the basal Ca^2+^ levels, r-irisin increased [Ca^2+^]_i_ approximately 4-fold and 6-fold at 10 and 50 nM, respectively (Fig 3A, 3B and 3D). Stimulation of cells with 20 mM KCl further increased [Ca^2+^]_i_ treated with 10 nM r-irisin (Fig 3A), but not at 50nM or 150nM (Fig 3B and 3C).

### R-Irisin Upregulated H9C2 Cellular Mitochondrial Metabolism

**Fig 4:**
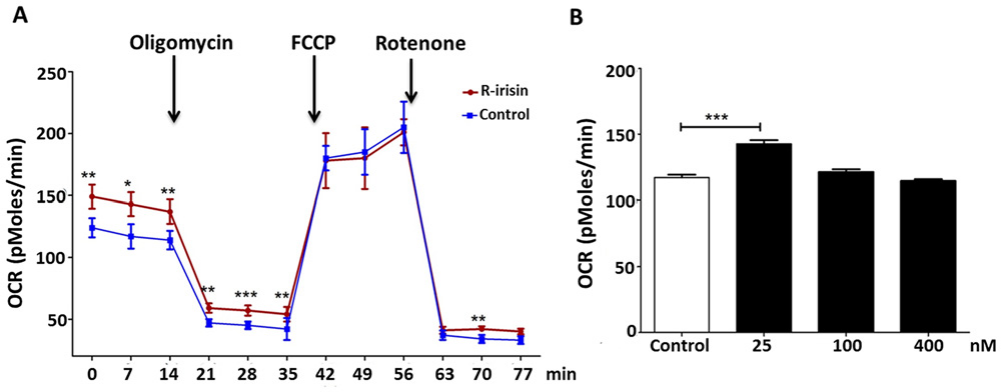
Effects of r-irisin on cellular oxygen consumption. A: Effects of r-irisin on cellular OCR was measured with the XF96 Extracellular Flux Analyzer. H9C2 cells were incubated with control (the supernatant of transformed with empty expression vector yeast cells) (blue line) or 25 nM (red line) of r-irisin for 72 h. Three inhibitors, oligomycin (1 μM), FCCP (0.75 μM) and rotenone (1 μM), were added at the indicated points. B: Effect of different r-irisin concentrations on H9C2 baseline OCR. H9C2 cells were incubated with control (the supernatant of transformed with empty expression vector yeast cells) or r-irisin 25 nM, 100 nM and 400 nM for 72 h. Results represent the means of eight independent experiments carried out in triplicate. (*, **, and *** indicating *p*<0.05, *p*<0.01, and *p*<0.001 statistical differences compared to control, respectively.)

Mitochondria supply the energy for a variety of cardiac functions, and cellular consumption of oxygen mainly reflects mitochondrial metabolism. To assess the effect of irisin on cardiomyoblast energetics, we measured OCR of H9C2 cells treated with different concentrations of r-irisin (or negative control) for 72 h using the Seahorse XF96 Extracellular Flux Analyzer. As shown in Fig 4A, 25nM r-irisin significantly increased baseline OCR values. However, higher concentrations of r-irisin (100 and 400 nM) showed no significant increase compared with the control (Fig 4B). To dissect the metabolic pathway leading to the OCR increase, oligomycin (1μg/ml) was added as an ATP synthase inhibitor. Inhibition of ATP synthesis by oligomycin significantly reduces electron flow through the electron transport chain [30]. The decrease in oxygen consumption rate upon injection of the oligomycin therefore represents the portion of basal respiration used to drive ATP production. Under this condition, OCR of H9C2 cells treated with r-irisin and the negative control was reduced to about 30% of baseline, indicating that about two-thirds (68%) was due to oxygen consumption linked to ATP production (Fig 4A). Strikingly, OCR improvement by r-irisin was reduced by oligomycin, although its value remained higher than in control cells. This result suggested that increasing ATP synthesis was also one function of r-irisin signaling. Next, we examined whether r-irisin affected mitochondrial maximum respiration. FCCP uncouples mitochondrial oxidative phosphorylation by dissipating membrane potential that drives ATP synthesis, and is thus commonly used to examine maximum respiration. Treatment with FCCP (0.75 μM) dramatically improved OCR values of H9C2 cells treated with r-irisin or negative control (Fig 4A). However, OCR differences between the two groups were not detected. Furthermore, incubation with 1 μM of the Complex I inhibitor, rotenone, induced a more glycolytic phenotype; OCR was reduced to about 20% of the baseline, representing non-mitochondrial respiration (Fig 4A). This result suggests that ~85% of the mitochondrial respiration was coupled to ATP synthesis and the rest was associated with proton leak. The higher OCR of H9C2 cells treated with r-irisin vs. control may indicate a potential up-regulation of the glycolysis pathway. Collectively, these data demonstrated that irisin increased mitochondrial metabolism in cardiomyoblasts.

### R-Irisin Activated Multiple Signaling Pathways in Myocardium of Mice

**Fig 5:**
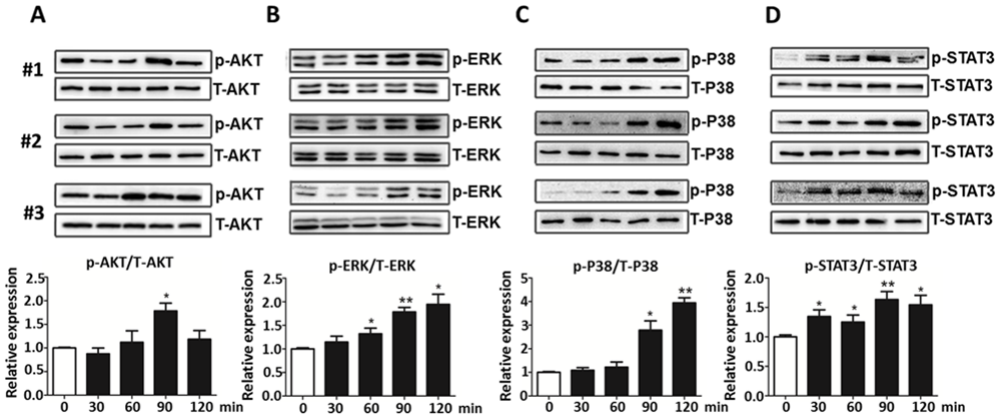
Activation of different signaling pathways in the mouse myocardium after r-irisin treatment. Male C57BL/6 mice were intraperitoneally injected with r-irisin (10 μg/g body weight) or control (the supernatant of transformed with empty expression vector yeast cells) (n = 3/group). After injection, the myocardium was harvested after 0, 30, 60, 90, and 120 min and cellular proteins were extracted from the left ventricles for western blotting. Total AKT, ERK, p38-MAKP and STAT3 protein served as protein-loading control. Top is typical blots from three independent group; the bottom bar graphs are the densitometry analyses for p-AKT/T-AKT (A), p-ERK/T-ERK (B), p-p38/T-P38(C) and p-STAT3/T-STAT3(D). All data were normalized against the control group. (* and ** indicating *p*<0.05 and *p*<0.01statistical differences compared to control, respectively.)

The above experiments using H9C2 cells clearly demonstrated the importance of irisin for cardiomyoblasts metabolism and cell growth/differentiation. Next, we investigated the *in vivo* effect of irisin on cardiomyocytes. Male C57BL/6 mice were injected with r-irisin (10 μg/g body weight i.p.) or the same volume supernatant of yeast transformed with an empty expression vector (n = 3/group). Myocardial tissues were then harvested and snap-frozen in liquid N_2_ at 0, 30, 60, 90, and 120 min. Cellular proteins were extracted from left ventricles to examine cell signaling protein expression by western blotting (Fig 5). We observed increased levels of phosphorylated AKT at 60 min, which was then decreased after 120 min. Interestingly, r-irisin also activated ERK, p38 and STAT3 signaling *in vivo* (Fig 5B–5D). The STAT3 signaling pathway was activated within 30 min (p<0.05), whereas ERK-MAPK and p38-MAPK were activated at 60 and 90 min, respectively. All three pathways showed phosphorylation up to 120 min (Fig 5B–5D). Given the importance of these pathways in heart health, the animal studies thus suggested that irisin may serve as a mediator of exercise-associated cardiac benefits.

### Hr-Irisin from HEK 293 Cells Had Similar Biological Activities

**Fig 6:**
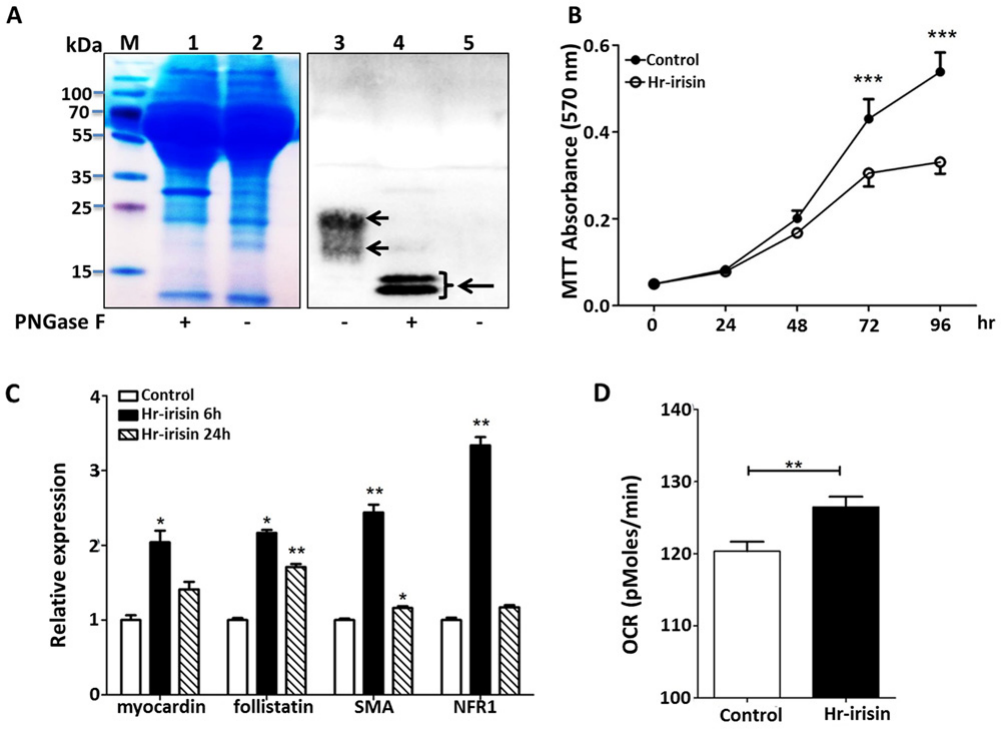
Effects of hr-irisin on cell growth. A: SDS-PAGE and western blotting analysis of human irisin expressed in HEK 293 cells (hr-irisin). The culture medium of irisin transfected cells was harvested for analysis. In Coomassie blue stained SDS—PAGE, Lane 1: medium supernatant containing hr-irisin; Lane 2: medium supernatant containing hr-irisin incubated with PNGase F; and M: Marker. In western blotting analysis, Lane 3: medium supernatant containing hr-irisin; lane 4: medium supernatant containing hr-irisin incubated with PNGase F; and Lane 5: medium supernatant of GFP transfected HEK 293 cells. Anti-irisin rabbit antibody was used in western blotting analysis. B: The time response of H9C2 cells to hr-irisin treatment. Cell proliferation was measured by MTT assay. C: Expression profile of selected genes in H9C2 cells treated with hr-irisin at different time points. Gene transcription was measured by quantitative RT-PCR, and compared to the expression level of β-actin. D: Effects of hr-irisin on H9C2 baseline OCR. H9C2 cells were incubated with control (GFP-containing medium) or same volume hr-irisin for 72 h and then measured with the XF96 Extracellular Flux Analyzer. In all studies, the data were shown as mean ±SD of three independent experiments. (*, **, and *** indicating p<0.05, p<0.01, and p<0.001 statistical differences compared to control, respectively.)

Yeast has different glycosylation modifications with mammalian cells. To more comprehensively understand the potential functions of irisin in animals and humans, we produced hr-irisin by transfecting HEK293 cells with irisin- or GFP-containing (negative control) plasmids. The culture medium was then analyzed by SDS-PAGE and western blotting using anti-irisin antibodies (Fig 6A). Although the bands for hr-irisin from SDS-PAGE were broad, probably due to glycosylation, (Fig 6A, lanes 1 and 2), western blotting confirmed the presence of hr-irisin protein (Fig 6A, lane 3). The majority of hr-irisin was highly glycosylated at both sites, which is slightly different with r-irisin (Fig 1A, lanes 1 and 2). Removal of the sugar moieties from hr-irisin with PNGase F (Peptide-N-Glycosidase F) resulted in two closely migrating bands of Mr ~ 12–15 kDa (Fig 6A, lane 4). A single band of Mr ~ 12–15 kDa was previously found in western blotting analysis of r-irisin treated with PNGase F [8]. No irisin was detected in the GFP-transfected control medium (Fig 6A, lane 5). To examine the biological activity of hr-irisin, we treated H9C2 cells with hr-irisin-containing medium (50 μl into 200 μl volume) or the GFP negative control. Similar to r-irisin, hr-irisin substantially inhibited H9C2 cell proliferation after 48 h (Fig 6B), and activated multiple genes related to cardiac function (Fig 6C). Hr-irisin also increased OCR baseline values in H9C2 cells (Fig 6D). Although it is difficult to determine the precise hr-irisin concentration in the HEK293 medium and the amount of hr-irisin used is lower than the detectable level of SDS-PAGE analysis, the similar effects of hr-irisin and r-irisin on cardiomyoblast growth and mitochondrial thermogenesis provided an important foundation for investigating the role of this myokine in heart health and disease.

### Detection of R-Irisin-His Binding to Cell Membrane of Cardiomyoblasts

**Fig 7:**
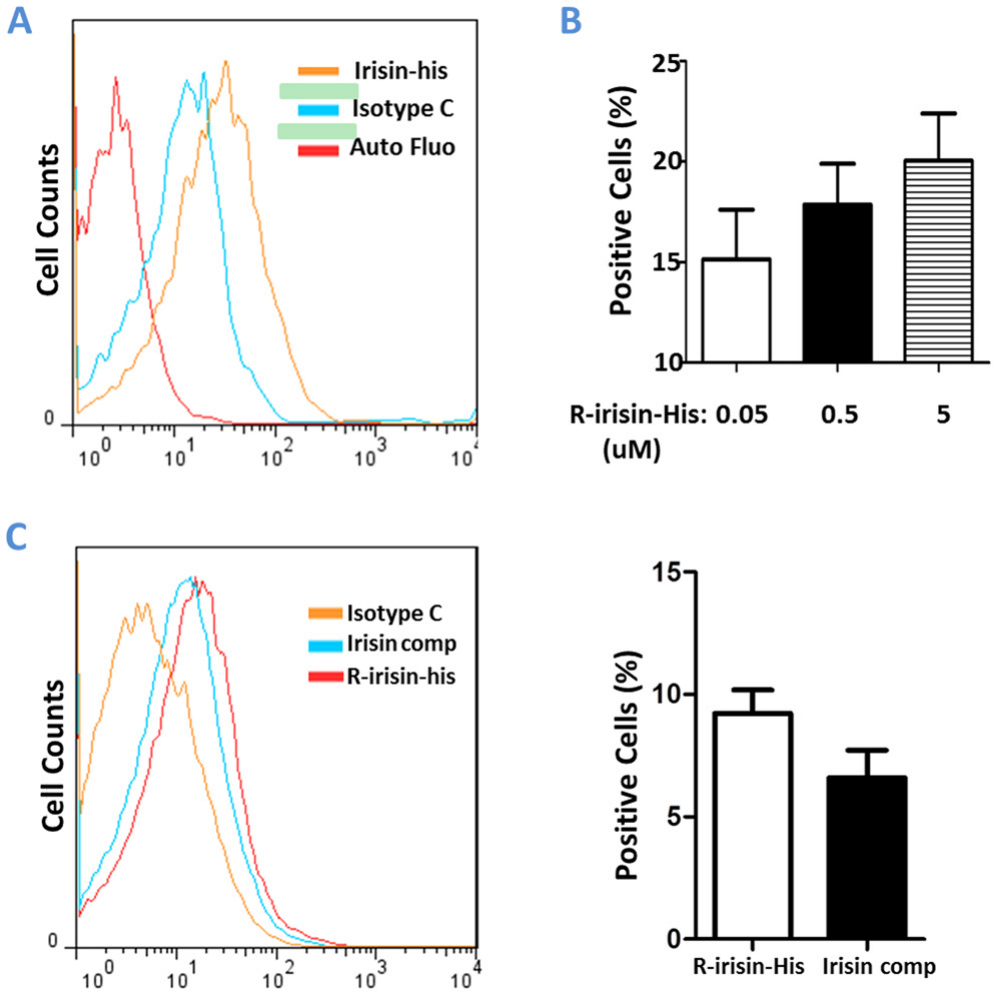
Detection of r-irisin-his binding to cell membrane of H9C2 cells. A&B: Detection of irisin binding to cell surface by flow cytometry. Flow cytometry using anti-his-PE antibody detected r-irisin-his that bound to H9C2 cells. Cells were pre-incubated with 5 uM r-irisin-his for 1h. A: Intensity of fluorescence in histogram of flow cytometry. Red is auto-fluorescence of H9C2 cells (red), blue for isotype control, and orange line for irisin-bound cells (r-irisin-his:anti-His-PE-positive). B: R-irisin binding to H9C2 cells was dose dependent. C: R-irisin competed with r-irisin-his in binding to H9C2 cells. H9C2 cells were incubated with r-irisin-his (50nM) or both r-irisin (50μM) and r-irisin-his (50nM) for 1h. Green line, isotype control; Orange line, r-irisin and r-irisin-his competition; Blue line, r-irisin-his only; Red line, auto fluorescence. The same results were expressed in bar graph (right panel).

To generate more insights into irisin signaling in H9C2 cells, we investigated the presence of putative irisin receptors on the cell membrane. We first expressed and purified r-irisin-his with the His tag appended on its C-terminus. The biological activity of r-irisin-his protein was validated by activating its known target genes. R-irisin-his and r-irisin showed similar biological activity in upregulating UCP1 expression in 3T3-L1 cells and myocardin, follistatin, SMA and NRF1 expression in H9C2 cells (S1 Fig), indicating that the engineered His tag at its C-terminus induced no-to-minimal effects on irisin functions. Next, we examined whether r-irisin-his binds to the H9C2 cell membrane using flow cytometry. As shown in Fig 7A, the numbers of anti-His-PE: r-irisin-his positive cells were markedly increased after the incubation of H9C2 cells with r-irisin-his compared with the isotype controls. The increase was dose dependent (Fig 7B). To examine whether the His tag in r-irisin-his contributed to the observed binding, we used r-irisin to compete with r-irisin-his in binding to H9C2 cells. As shown in Fig 7C, the numbers of anti-His-PE: r-irisin-his positive cells were decreased to the isotype control level when r-irisin concentration was 1000 times higher than r-irisin-his. These results suggest that the r-irisin moiety is involved in the binding, and yet-to-be-identified irisin receptor(s) are present on the membrane of H9C2 cells.

## Discussion

Skeletal muscle is a dynamic tissue, the complexity of which is only partially understood. Irisin, a novel myokine, is a cleavage product of FNDC5 and is produced in response to exercise [5] or cold exposure [31]. In their seminal paper, Boström et al. demonstrated that irisin increases UCP1 expression leading to the browning of white adipocytes [5]. Given its potential role in mediating exercise benefits and its exciting promise in obesity management, numerous studies have focused on the characterization of irisin’s physiological functions and its relevance to human health. For example, exercise is well-known for its beneficial effects on the heart, one organ with significant irisin production [10]. Furthermore, several studies have suggested a potential role for irisin in CVD [12–15]. In this report, we provide new insight into the mechanism by which irisin may have beneficial effect on cardiovascular system on animals and on cardiomyoblast function.

As an exercise-derived hormone, irisin directly passes signal to heart or other tissues. It is therefore important to identify the molecular pathways mediating beneficial effects of irisin in cardiomyoblast. Irisin binding study supports the existence of irisin-specific receptor on the cell surface of H9C2 cells (Fig 7). Through this receptor, irisin may activate several downstream signaling pathways in cardiomyoblasts (Figs 2 and 5). P38-MAPK and ERK are implicated in a wide range of cellular processes, from cell growth and proliferation to apoptosis [32]. Previously, we reported the involvement of p38-MAPK and ERK pathways in irisin mediated browning of mouse/rat adipocytes [8]. We also demonstrated that the ERK signaling is necessary for the proliferation of human umbilical vein endothelial cells [31]. These pathways are also important for heart development, remodeling and metabolism [26, 27]. Interestingly, our studies indicated irisin did not activate these pathways in H9C2 cells. Instead, phosphorylation of AKT was found in r-irisin treated H9C2 cells and also observed in the myocardium of mice injected with r-irisin. AKT activation is regulated by a number of factors like insulin, exercise training, pressure overload or even disease [33]. The PI3K-AKT pathway is critical for cardiomyocyte growth and function [25]. These results suggest that irisin might exert its effects (growth, thermogenesis, etc.) on H9C2 cells through the regulation of the PIK-AKT pathway. In contrast with the results from the cell model, ERK, p38-MAPK, and STAT3 were also activated after injecting r-irisin into mice. The complex nature of mice model might lead to this discrepancy. For example, once irisin reaches the circulation system of mice, we suspect that it may impact biological processes of multiple organs/tissues of mice (e.g. muscle, adipose)[8, 34]. Consequences of these simultaneous effects are unknown but may lead to the activation of ERK, p38-MAPK, and STAT3 signaling pathways in myocardium. It is also possible that irisin may exhibit cross-talk with other poorly-characterized myokines produced by skeletal muscle of mice [4] to exert its effect. Further studies are required to clarify the observed discrepancy between two models and dissect the importance of these pathways during irisin stimulation.

Another important finding of this work was irisin’s ability to increase intracellular Ca^2+^. Calcium signaling is required for the functioning of cardiac muscle cells where it is actively involved in contraction and electrical conductivity. One beneficial effect of exercise is the improvement of cardiac excitation—contraction coupling through Ca^2+^ signaling [29]. The fact that irisin increased intracellular Ca^2+^ in H9C2 cells at 10 and 50 nM suggests a potential role for this myokine in cardiac contractility. However, irisin failed to increase intracellular Ca^2+^ at 150 nM or greater concentrations. It is possible that inhibitory pathways (e.g. Gi/Go protein activation) might be activated by high irisin concentrations [35, 36]. This could explain the lack of response to 150 nM irisin and 20 mM KCl (Fig 3C). In consistent with this result, high-dose irisin failed to further increase the oxygen consumption rate as well. In addition, multiple signaling pathways including PI3K-AKT regulate Ca^2+^ dynamics [37, 38]. The PI3K-AKT pathway was a key in mediating irisin effects as shown in this study. At higher concentrations of irisin, this pathway might also play a role in inhibiting the intracellular Ca^2+^ signals such as phosphorylation of key proteins [39]. Indeed, several ion channels and Ca^2+^-dependent proteins (e.g. calmodulin) that mediate the increases of intracellular Ca^2+^ are inhibited by phosphorylation events [40, 41]. The precise mechanism underlying Ca^2+^ signaling during irisin stimulation of cardiac muscle cells remains to be determined.

In our results, r-irisin up-regulated the expression of key genes associated with muscle growth and differentiation. Myocardin is a master regulator for smooth muscle [21] that mediates postnatal cardiac growth and remodeling [22]. Follistatin is released after acute exercise and promotes muscle growth via inhibiting myostatin [23]. Moreover, SMA initiates cardiomyocyte differentiation and cardiac growth [24]. Up-regulation of these genes in H9C2 cells by r-irisin suggests that it may stimulate cardiac growth and differentiation. It has been shown that the AKT pathway regulates the expression of follistatin in skeletal muscle [42], myocardin release [43] and SMA expression [44]. Recently, Li et al. also showed that Ca²⁺signaling can activate the expression of myocardin [45]. Therefore, the observed changes of gene transcription profiles may be regulated by the PI3K-AKT pathway and calcium signaling, both of which were activated by irisin in this report.

As a mitochondria-rich tissue, the heart has a high rate of O_2_ consumption and ATP production/turnover that is required to maintain its continuous mechanical work, especially after exercise. Interestingly, increased oxygen consumption was observed in H9C2 cells after incubating with 25 nM r-irisin, but no further increase in oxygen consumption was observed with higher concentrations of r-irisin (100 nM and 400 nM), consistent with our findings from Ca^2+^ imaging experiments. These data suggest that irisin may control myocardial contraction and metabolism during exercise at low concentrations. In adaptive thermogenesis, the increase in mitochondrial respiration can be driven by uncoupling electron transport from ATP synthesis. Using the ATP synthesis inhibitor oligomycin, we found that irisin improved mitochondrial thermogenesis mainly via heat production but also via ATP synthesis. PGC-1α is essential for cellular responses during exercise, starvation and cold [7]; its overexpression leads to energy dissipation as heat [6]. After expression, PGC-1α activates specific transcription factors involved in cellular metabolism, such as NRF1 [6]. NRF1 is important for replication, maintenance, and transcription of mitochondrial DNA and controls the expression of respiratory chain subunits and other proteins required for mitochondrial function [24]. When H9C2 cells were treated with 50 nM r-irisin, transcription levels of NRF1 increased more than 2-fold. Therefore, irisin might also regulate mitochondrial thermogenesis. Interestingly, the PI3K-AKT pathway also activates the NRF1 transcription under oxidative conditions [37]. However, the uncoupler FCCP failed to differentiate H9C2 cells under tested conditions, which suggests additional studies to examine irisin’s role in mitochondrial biogenesis.

Previous studies have shown the presence of irisin in the human body [46–48], but its expression and molecular weight in mammalian cells are still controversial. For example, Albrecht et al. [9] have raised questions about the physiological roles of irisin in humans and other species. The therapeutic potential of irisin to fight obesity and diabetes calls for further research to investigate its potential benefits. Human irisin expressed in HEK293 cells deglycosylation generated two closely migrating bands of 12–15 kDa, similar to the reported values [5, 49]. R-irisin produced in yeast also exhibited three bands with mobility of ~12, 17 and 22 kDa. The differences may be due to the sugar moieties used by yeast and mammalian cells in protein glycosylation [50]. Yeast generally glycosylates proteins with high-mannose (with more than 3 mannose residues) or hypermannose (more than 6 mannose residues), which potentially reduces the half-life of glycosylated proteins, compromising therapeutic activity [51]. Due to the low levels of hr-irisin in the culture medium, it is difficult to accurately determine its concentration in the sample. Nevertheless, hr-irisin and r-irisin had similar effects on H9C2 cell growth. It appears that hr-irisin has a greater inhibitory effect on H9C2 cell proliferation and is more effective in regulating gene transcription. We have previously done direct site-mutation on the two glycosylation sites of irisin and found that the posttranslational glycosylation of the secreted irisin from yeast had an effect on the biological function of the browning effect [8]. These data suggest that different glycan moieties may account for the potency difference. In general, the yeast system is more advantageous because it offers a large quantity of active r-irisin for biological, preclinical, and even clinical tests.

## Conclusions

In summary, the recently discovered myokine irisin may be important for proper cardiac function. Our data showed that irisin inhibited H9C2 cell proliferation but enhanced its growth. Furthermore, we found that the PI3K-AKT pathway was activated by irisin, which might be linked to the observed cell growth. In the mouse model, this pathway as well as the ERK, p38-MAPK and STAT3 pathways was activated in the myocardium after injection of r-irisin. Additionally, we observed an increase in intracellular Ca^2+^ and mitochondrial thermogenesis in a certain concentration range of irisin. Our *in vitro* and *in vivo* results demonstrate for the first time the importance of irisin for cardiac function. The results also support the existence of irisin-specific receptor on the membrane of H9C2 cells. Additional experiments are needed to characterize the effects of irisin on primary cardiomyocytes and in clinically relevant animal models.

## Supporting Information

S1 Fig R-irisin-his protein has compatible biological activity as r-irisin. A: Irisin-mediated UCP-1 in mouse 3T3-L1 adipocytes. Cells were treated with indicated two types of r-irisin at 25 nM for 24h and UCP-1 expression was examined by qRT-PCR as previously described [8]. B: Expression profile of selected genes in H9C2 cells treated with r-his-irisin at 50 nM for 6hs. The gene expression was measured by qRT-PCR. The data was shown as mean ±SD of three independent experiments. * and **represent p<0.05, p<0.01, respectively. These two experiments show similar biological activity in the native r-irisin and his-tagged r-irisin-his.
(TIF)

S1 Table Real-Time PCR Primers of cardiomyoblasts related genes. (DOCX)
