# Supplementary figures and images for: Irisin Controls Growth, Intracellular Ca2+ Signals, and Mitochondrial Thermogenesis in Cardiomyoblasts
Source: PLoS One. 2015 Aug 25;10(8):e0136816. doi: 10.1371/journal.pone.0136816 (PMC4549318; doi:10.1371/journal.pone.0136816)

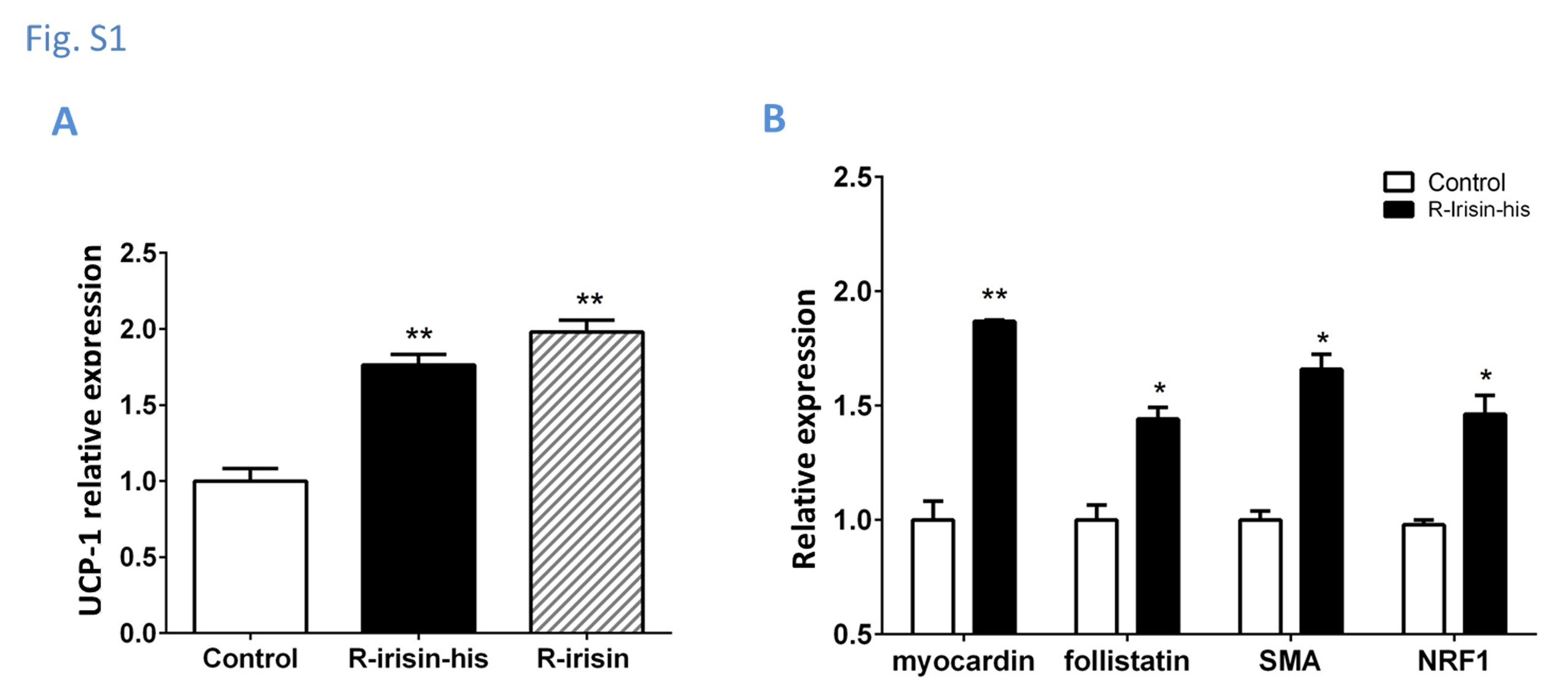

Supplement: S1 Fig — A: Irisin-mediated UCP-1 in mouse 3T3-L1 adipocytes. Cells were treated with indicated two types of r-irisin at 25 nM for 24h and UCP-1 expression was examined by qRT-PCR as previously described [8]. B: Expression profile of selected genes in H9C2 cells treated with r-his-irisin at 50 nM for 6hs. The gene expression was measured by qRT-PCR. The data was shown as mean ±SD of three independent experiments. * and **represent p<0.05, p<0.01, respectively. These two experiments show similar biological activity in the native r-irisin and his-tagged r-irisin-his. (TIF) [file pone.0136816.s001.tif]
