# Supplementary material for: Irisin Controls Growth, Intracellular Ca2+ Signals, and Mitochondrial Thermogenesis in Cardiomyoblasts
Source: PLoS One. 2015 Aug 25;10(8):e0136816. doi: 10.1371/journal.pone.0136816 (PMC4549318; doi:10.1371/journal.pone.0136816)
Supplement: S1 Table — (DOCX) [file pone.0136816.s002.docx]

**Supporting Information**

**Figure S1. R-irisin-his protein has compatible biological activity as r-irisin.** **A**: Irisin-mediated UCP-1 in mouse 3T3-L1 adipocytes. Cells were treated with indicated two types of r-irisin at 25 nM for 24h and *UCP-1* expression was examined by qRT-PCR as previously described [1]. **B:** Expression profile of selected genes in H9C2 cells treated with r-his-irisin at 50 nM for 6hs. The gene expression was measured by qRT-PCR. The data was shown as mean ±SD of three independent experiments. * and **represent *p<0.05, p<0.01*, respectively. These two experiments show similar biological activity in the native r-irisin and his-tagged r-irisin-his.


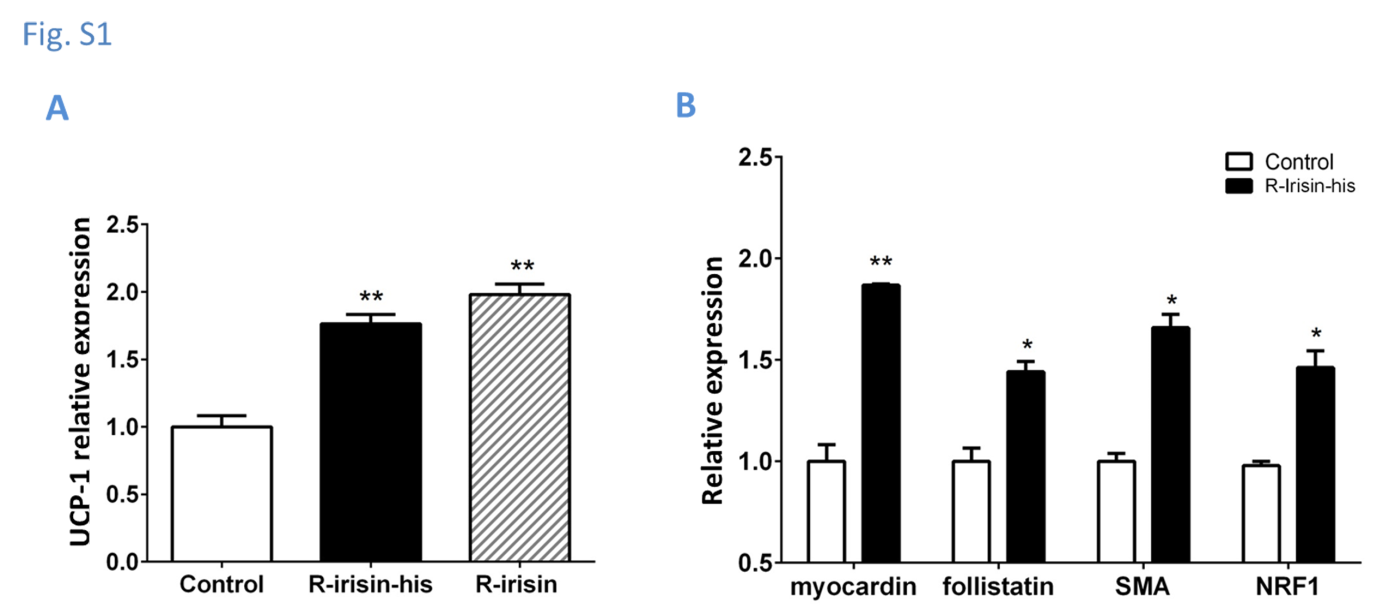


**Table S1. Real-Time PCR Primers of cardiomyoblasts related genes.**

| **Gene** | **Accession No.** | **Type** | **Sequence(5’-3’)** |
| --- | --- | --- | --- |
| **SMA** | NM_031004.2 | Sense | TCCTGACCCTGAAGTATCCGATA |
|  |  | Anti-sense | GGTGCCAGATCTTTTCCATGTC |
| **myocardin** | NC_005109.3 | Sense | GCTGAGCAACCAGTCAGACTC |
|  |  | Anti-sense | GTAGCTGCAGGAATAGCTGC |
| **follistatin** | NC_005101.3 | Sense | CGTGGACCGAGGAGGATGTG |
|  |  | Anti-sense | CAGGTGATGTTGGAACAGTC |
| **NRF1** | NM_031789.2 | Sense | GCCAGCTGAACTCCTTAGAC |
|  |  | Anti-sense | GATTCGTGCACAGCAGCA |

Reference List

1. Zhang Y, Li R, Meng Y, Li S, Donelan W, Zhao Y, Qi L, Zhang M, Wang X, Cui T, Yang LJ, Tang D (2014) Irisin stimulates browning of white adipocytes through mitogen-activated protein kinase p38 MAP kinase and ERK MAP kinase signaling. Diabetes 63: 514-525. db13-1106 [pii];10.2337/db13-1106 [doi].
